# Mental health among students with neurodevelopment disorders: case of dyslexic children and adolescents

**DOI:** 10.1590/1980-57642021dn15-040014

**Published:** 2021

**Authors:** Said Ihbour, Hammou Anarghou, Abdelmounaim Boulhana, Mohamed Najimi, Fatiha Chigr

**Affiliations:** 1 Biological Engineering Laboratory, Faculty of Science and Techniques – Beni Mellal, Morocco.

**Keywords:** self-esteem, anxiety, depression, dyslexia, autoestima, ansiedade, depressão, dislexia

## Abstract

Several research studies have been devoted to study the links between emotional disorders and learning disabilities. However, very minimal of this research has focused on dyslexic students.

**Objective:**

The objectives of this study were as follows: (1) to assess self-esteem, anxiety, and depression in dyslexic Arabic-speaking children and adolescents and (2) to describe psychiatric comorbidities in these subjects by comparing them to their non-dyslexic peers.

**Methods:**

In total, 205 students (56 dyslexics and 149 good readers), pursuing their education in ordinary schools in the Beni Mellal-Khenifra region of Morocco responded to Taylor’s Self-Assessment Scale of Anxiety, Beck’s Depression Questionnaire, and the Coopersmith Self-Esteem Inventory (SEI).

**Results:**

Overall, dyslexics were more anxious, more depressed, and had disturbed self-esteem compared to their non-dyslexic peers. The percentage of psychiatric comorbidity was higher in the dyslexic group.

**Conclusions:**

The results of this study highlight the need for a multidisciplinary approach that integrates emotional needs assessment into the rehabilitation care of dyslexic children and adolescents.

## RESUMO

Muitos estudos têm pesquisado as ligações entre transtornos emocionais e dificuldades de aprendizagem. No entanto, muito pouco desta pesquisa se concentrou em estudantes disléxicos.

**Objetivo:**

Os objetivos deste estudo foram: 1) avaliar a autoestima, ansiedade e depressão em crianças e adolescentes disléxicos falantes do árabe; 2) descrever comorbidades psiquiátricas nesses estudantes, comparando-os com seus pares não disléxicos.

**Métodos:**

205 estudantes (56 disléxicos e 149 bons leitores), alunos de escolas comuns da região de Beni Mellal-Khenifra do Marrocos, responderam à escala de autoavaliação de ansiedade de Taylor, ao questionário de depressão de Beck’s e ao inventário de autoestima de Coopersmith (IES).

**Resultados:**

Em geral, os disléxicos eram mais ansiosos, mais deprimidos e tiveram distúrbios de autoestima em comparação com seus pares não-disléxicos. O percentual de comorbidade psiquiátrica foi maior no grupo disléxico.

**Conclusões:**

Os resultados deste trabalho evidenciam a necessidade de uma abordagem multidisciplinar que integre a avaliação das necessidades emocionais aos cuidados de reabilitação de crianças e adolescentes disléxicos.

## INTRODUCTION

Several research studies have been devoted to study the links between emotional disorders (anxiety, depression, and low self-esteem) and learning disabilities, and it is only some of these works that have been aimed at dyslexic children and adolescents. Although the trend emerging from these different researches confirms that dyslexics seem to have more psycho-emotional disorders, the results were contradictory. Some studies indicate a high prevalence of these disorders in children with this cognitive disability, with a significant overlap between the two pathology classes, while others do not find any difference between the subjects with and without reading difficulties. Thus, the study by Hoy et al.,^1^ Carroll and Iles,^2^ Grills-Taquechel et al.,^3^ Daviss,^4^ and Maag and Reid^5^ provided evidence that students with reading difficulties had more anxiety symptoms than their peers without difficulty. Similarly, meta-analyses have shown that children with this learning disorder have more depressive and anxious symptoms compared with the general population of children.^6^

These findings are consistent with the study by Maugham et al.^7^ who revealed that children with reading difficulties are significantly more depressed than their peers without difficulties. By analyzing the association between internalized disorders and learning disabilities, other researchers have arrived at the same results by finding that dyslexic students have an increased rate of psychopathological problems than non-dyslexic students.^8^ ^,^ ^9^ However, the study by Riddick et al.^10^ and also Mattek and Wierzbicki^11^ confirmed that the two groups (dyslexic and non-dyslexic) did not have considerable differences in the measures of depression symptoms. Similarly, the results of some self-esteem studies have also shown that dyslexic children and adolescents may have alterations in this mental health indicator^12^ while others claim that the self-esteem of these children does not differ significantly from that of their peers without learning disabilities.^13^

Despite the considerable number of research currently available on the links between psycho-emotional difficulties and learning disabilities in general, the scientific landscape shows a lack of work that simultaneously takes into account several psychological dimensions of dyslexic children and adolescents. The scientific literature also points to a knowledge gap related to the mental health of learners from different writing systems. Furthermore, unlike the so-called “opaque orthographic systems,” transparent languages generate less painful learning to read and write, which is likely to have less of an impact on mental health. In the same context, previous studies have shown that the prevalence of dyslexia varies from 2.3 to 12% depending on the transparency of the language and the definition is taken into account.^14^ ^,^ ^15^ ^,^ ^16^ ^,^ ^17^ For the Arabic language, the orthographic structure has two writing systems: one vocalized and the other unvocalized. In the first system, the presence of diacritical marks creates strict correspondences between graphemes and phonemes and thus provides unambiguous phonological and semantic information. In the unvocalized form of writing, only consonants and long vowels are transcribed, and this implies that the word is phonologically and semantically ambiguous, so the reader must appeal to the context to read correctly.^18^ ^,^ ^19^ In Morocco, as in many Arab countries, the notion of dyslexia as a biological pathology that specifically hinders reading acquisition is still ignored. Dyslexic disorders are not well known by Moroccan teachers because they were not part of their training programs, they are also poorly appreciated by doctors and paramedics. At the highest level of the institutions responsible for the educational system, the Supreme Council for Education, in the new strategic vision of reform (2015–2030), does not yet recognize the existence of dyslexic disorders. In the absence of the data based on the larger cohorts of children such as those in Anglophone and Francophone settings, a recent study that was conducted with a relatively large sample of elementary- and middle school students showed that the prevalence of dyslexia was 5.4%.^20^

The lack of knowledge of dyslexia, by those involved in the field of education and health, in Moroccan schools, makes it necessary to question the emotional well-being of children with this cognitive disability. In this context, our study aims, on the one hand, to assess the level of anxiety, depression, and self-esteem in dyslexic students by comparing them to their peers without dyslexia and, on the other hand, the analysis of correlations between the three indicators in the two groups of students. The originality of this research lies in the data from the Arabic-speaking subjects.

## METHODS

### General methodology

A total of 205 students (104 boys and 101 girls), pursuing their schooling in public primary and secondary schools in the Beni Mellal-Khenifra region in central Morocco, participated in this study. Among this sample, 56 subjects were previously identified as having reading difficulties evoking a developmental dyslexia profile, and they constitute the dyslexic group. The other 149 are classified as normal readers and represent the “control” group. The identification abilities of isolated words in Arabic (e.g., simple and plural words without diacritics and pseudowords with and without diacritics) were tested by subtests extracted from the LABEL software (Language assessment battery),^21^ which is a battery of tests for cognitive language assessment for Arabic-speaking people. The accuracy and speed of reading were measured by the 1-minute reading test of the Arabic vocalized words (Khomsi test) inspired by the studies of Ammar.^18^ The tests assessing reading performance were administered individually. The “normal-readers” group is composed of children who have succeeded in all or at least some of the tests. The profile of dyslexia has been identified by analyzing the nature and frequency of errors made by the student, the protocol is the same as the one applied in our previous study focused on the diagnosis of dyslexic disorders in Arabic-speaking Moroccan children.^20^ In some cases, school performance and teachers’ reports also helped us to easily target children with major reading difficulties to approach the diagnostic test for dyslexia. Students from both groups were paired, to the possible limits, on age and socioeconomic status of the parents. The population of the study is submitted to three self-assessment questionnaires measuring anxious symptomatology, depressed mood, and self-esteem level.

### Tools

#### Coopersmith Self-Esteem Inventory

To assess the overall self-esteem, we used the school version of the Coopersmith Inventory (SEI),^22^ which is a questionnaire that applies to children and adolescents in school and determines the degree of general self-satisfaction. The choice of this instrument is motivated by the place that it makes to the school field. It includes 58 items that cover four areas of the self-esteem measurement as follows: general, family, social, academic, and a lie index. These items describe feelings, opinions, or reactions of an individual order, to which the subject must answer by checking a box: “Looks like me” or “Does not look like me.” The scores in the various domains allow us to assess the extent to which subjects have a positive self-image. The total score of self-esteem is obtained by summing the scores on the four subscales (General, Family, Social, and School with a maximum score of 26 for the General scale and 8 for the other scales). The score obtained on the lying scale indicates the extent to which the subject has a defensive attitude towards the test. An overall score of less than 18 corresponds to low self-esteem.

#### The Beck Depression Inventory

The BDI^23^ is a self-assessment scale that gives a quantitative estimate of depression intensity. This scale is composed of 21 items, i.e., each item comprising 4 sentences corresponding to 4 degrees of increasing intensity of a symptom on a scale from 0 to 3. The subject is asked to complete the questionnaire by circling the number that corresponds to the chosen proposition. The total score is obtained by adding the different responses while taking into account the highest score for each series. This scale allows retaining the diagnosis of depression from a threshold of 10 points, the higher the score, the more the subject is depressed.

#### Taylor’s inventory of manifest anxiety

Taylor’s inventory of manifest anxiety^24^ is a questionnaire that explores the symptomatology of overt anxiety, and it consists of 50 statements each of which must be evaluated as true or false. Anxiety intensities are determined by the number of “true” responses. The threshold score is 17 (moderate to very severe anxiety symptomatology), the higher the total score the more anxious the subject is.

## RESULTS

### Characteristics of the sample and measurement tools

The “clinical” group consists of 56 students (41 boys and 15 girls) of average age: A=12.1 years (min=10 years; max=17 years; SD=1.87). The representatives of the “control” group, 63 boys and 86 girls, had an average age of A=11.71 years (min=9 years; max=16 years; SD=1.72). The average age of the first group is relatively high due to the repetition of school years. Regarding the reliability of the scales used, the index of internal consistency showed satisfactory values for all three scales (0.94 for SEI items, 0.86 for Beck’s depression questionnaire, and 0.89 for Taylor’s inventory of manifest anxiety). Correlations between the subscales of self-esteem range from 0.64 (family and social dimensions) to 0.85 (school and general components).

### Self-esteem

The average score of overall self-esteem differs significantly between subjects in the dyslexic group and those in the “normal-readers” group. In the first group, 82.2% of participants have a “low to very low” overall self-esteem against 16.7% in the second group. The percentages of students with average to very high self-esteem in both groups are, respectively, 17.9 vs. 83.3%. In practical terms, one student in two has very low self-esteem in the group with reading difficulties (≤18). In both study groups, there was a slight difference, statistically insignificant, in favor of boys (F=1.06, p=0.3 and F=2.7, p=0.1 successively in dyslexic patients and “normal-readers”).

The SEI shows that the different dimensions of self-esteem are disrupted in dyslexics. Thus, the scores obtained at the four subscales (i.e., general, academic, social, and family) of self-esteem are significantly higher among learners without dyslexia compared to their dyslexic peers (Table 1). In the latter group, the results do not reveal a significant difference by gender (even if boys’ scores are higher than those of girls) in the four fields. In contrast, among subjects without reading difficulties, boys had higher scores than girls in the family component (T=2.91; p=0.004), and lower scores in the school domain (T=2.38; p=0.018). These results must be interpreted with caution since the proportions of the two genders are different in the two groups (i.e., the clinical group vs. the control group). If we look at the relationships between the different dimensions of “self-esteem,” we find that the four evaluation domains constituting the SEI are positively correlated with each other in the two study groups (i.e., the correlation is significant at the 0.01 level). The intensity of this relationship is very strong in the “disabled” group (0.7<r<0.95 vs. 0.15<r<0.57). When looking at the variation of global self-esteem in the dyslexic group, significant correlations were found between the total self-esteem score and the scores obtained on different reading tests. These correlations mean that subjects with severe dyslexia had lower self-esteem than their peers with attitudes suggestive of a “mild dyslexia” profile.

**Table 1.** Self-esteem, anxiety, and depression in both study groups.

	Normal- readers (n=149)	Dyslexics (n=56) average (DS)	Student’s t-test	p-value
Self-esteem				
General subscale	18.66 (3.90)	8.39 (6.39)	−13.81	0.000
Social subscale	5.58 (1.62)	2.84 (2.41)	−9.3	0.000
Family subscale	5.89 (1.23)	3.68 (1.86)	9.8	0.000
School subscale	6.44 (1.79)	2.37(2.30)	11.8	0.000
Global self-esteem	36.60 (6.75)	17.29 (11.59)	11.35	0.000
Anxiety	17.97(8.57)	28.65(7.73)	−8.43	0.000
Depression	25.11 (13.69)	36.5 (7.52)	−7.02	0.000

### Study of anxiety and depression

**Figure 1.**
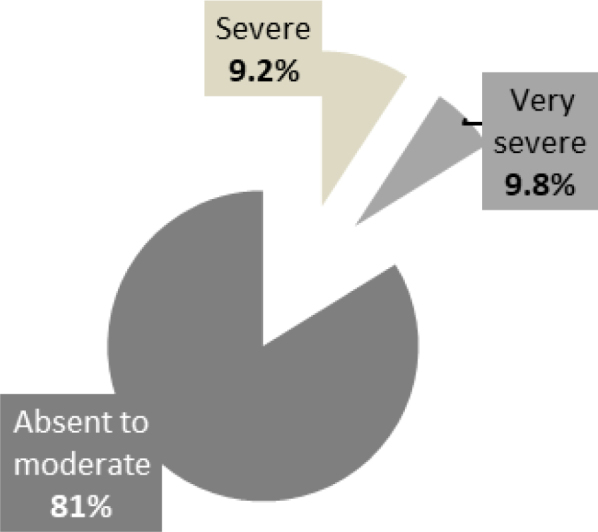
Intensity of anxiety symptomatology in the “normal-readers” group.

**Figure 2.**
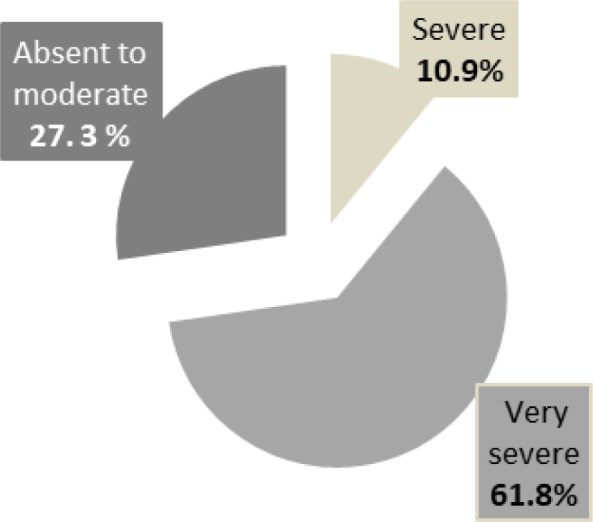
Intensity of anxiety symptomatology in the “dyslexic” group.

Taylor’s inventory of manifest anxiety that was used to evaluate anxiety symptomatology revealed that dyslexic students have a significantly higher average score of anxiety than “normal-readers” (Table 1). The intensity of anxiety symptoms in both groups is shown in Figures 1 and 2. Although the results do not reveal any significant differences by gender, the average scores of girls’ anxiety are higher than those of boys in both groups (i.e., girls are more anxious than boys).

**Figure 3.**
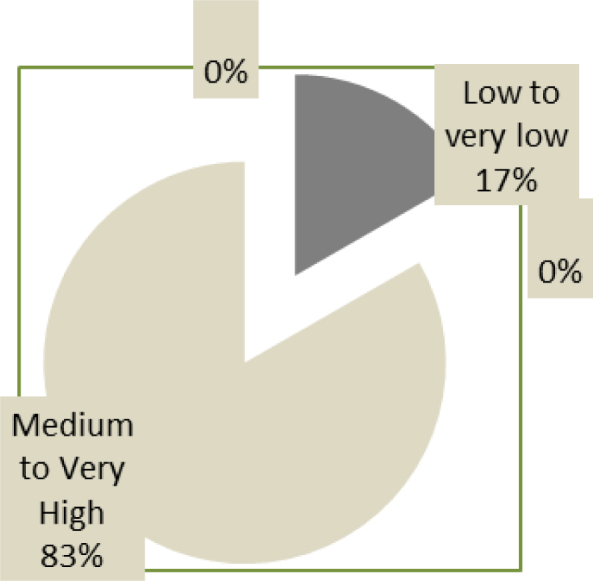
Overall self-esteem status in the “Normal-readers” group.

**Figure 4.**
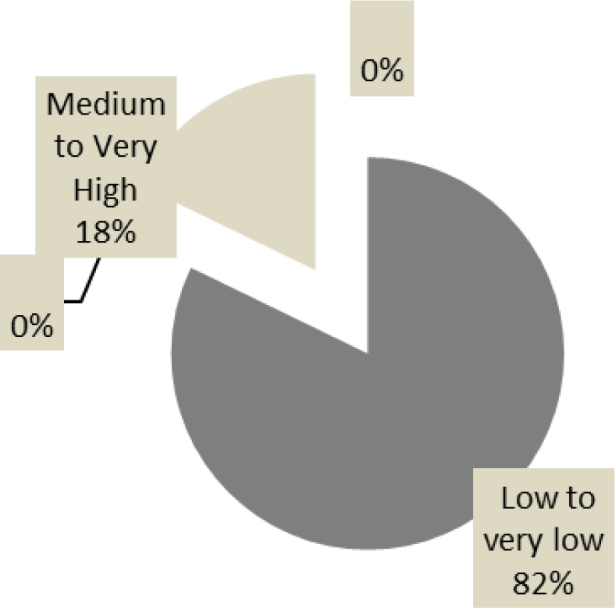
Overall self-esteem status in the “Dyslexic” group.

**Figure 5.**
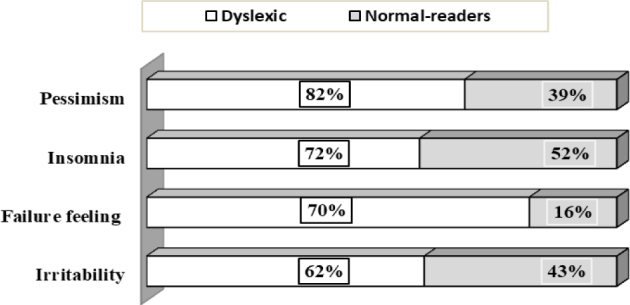
Results obtained for some items of Beck’s inventory in both groups.

As for depression, the Beck’s questionnaire found a very significant difference in the average scores between the two groups (Table 1). Dyslexics have a significantly higher depressive symptomatology than their “normal-readers” peers (T=7.02; p=0.000) (Figures 3 and 4). No relevant differences were recorded by gender in both groups. The study of some items of Beck’s inventory in the two groups is represented in Figure 5. When examining the impact of severity of dyslexia on the anxiety-depressive symptomatology, we found that the total scores of anxiety and depression correlated negatively with reading test scores in the dyslexic group. This means that “severe dyslexics” have more anxiety-depressive symptoms than their peers with “mild dyslexia.”

### Relationship between self-esteem, anxiety, and depression

The statistical analysis shows that the average score of overall self-esteem is negatively correlated, in a significant way, with depression and anxiety in the “normal-readers” group. In the “dyslexic” group, this correlation is only relevant between global self-esteem and anxiety (Table 2). When we analyzed that the relationships between self-esteem components and scores reflect the level of anxiety and depression, we observed that the four subscales of overall self-esteem (i.e., general, social, family, and academic) are negatively correlated (i.e., the correlation is relevant) with anxiety in both groups. In the case of depression, the correlation is significant only with the general, social, and academic subscales for the “control” group and only with the social dimension in the “clinical” group (Table 2). The study of the links between depression and anxiety also revealed a significantly positive correlation between these two indices of mental health in dyslexics and “normal readers.” Although these results do not specify the effect of causality, they show that the two psychopathological disorders evolve in the same direction within our population.

**Table 2.** Correlations between self-esteem, anxiety, and depression in the two study groups.

Groups	Normal-readers (n=149)	Dyslexics (n=56)
Self-esteem≠anxiety		
General scale≠anxiety	−0,641 **	− 0,62**
Social scale≠anxiety	−0,51**	−0,67**
School scale≠anxiety	−0,44**	−0,60 **
Family scale≠anxiety	−0,31**	−0,40 **
Global S-E≠anxiety	−0,66 **	−0,64 **
Self-esteem≠depression		
General scale≠depression	−0,57**	NS
Social scale≠depression	−0,44**	−0,28*
School scale≠depression	−0,38**	NS
Family scale≠depression	NS	NS
Global S-E≠depression	−0,55**	NS
Anxiety≠depression	0,66**	0,49**

Spearman correlation test: *p<0.05; **p<0.01.

Among dyslexic children and adolescents, the percentage of subjects with “severe to very severe” symptoms of anxiety and depression associated with “low to very low” self-esteem is higher than in the control group (62.5 vs. 5.63%, respectively). By analyzing the association between the age and scores obtained at the three scales in both groups, it appears that this association is only relevant between age and the overall self-esteem score in the group without reading difficulties (r=-2.41; p=0.004). Finally, the comparison between girls and between boys in both groups (dyslexic and non-dyslexic) indicates that group subjects with this developmental disorder are more depressed, more anxious, and have low self-esteem when compared to their “normal” peers.

## DISCUSSION

We remind that the objective of our study was to explore the anxiety, depression, and self-esteem of dyslexic children and adolescents schooled in ordinary educational establishments. This field, which has sparked less research in the Anglo-Saxon and Francophone world, remains unexplored in the Arabic-speaking countries. To our knowledge, no empirical study has explored the psycho-emotional disorders simultaneously on the three psychic dimensions in dyslexic students, hence the originality of this work both in the Arabic-speaking and in the international contexts.

The results of our study revealed a high frequency of depressive and anxiety disorders, associated with low levels of self-esteem, in dyslexic children and adolescents. These findings are consistent with those of several studies that have described dyslexics as much more affected by internalized emotional difficulties than their peers without dyslexia. Although the age of the participants, the site of recruitment, and the measurement instruments used are different for each study, their results converge towards the same conclusion describing a high frequency of psychiatric disorders with an internalized pace in dyslexic people. Thus, for the anxious symptomatology, the data resulting from our work corroborate those of Tsovili,^25^ Martinez and Sermrud-Clikeman,^26^ as well as those of Carroll and Islands.^2^ The first two studies concluded that dyslexic students appear to be more affected by state anxiety than their non-dyslexic peers, and the third leads to a high level of state anxiety in the social and academic components of the State-Trait Anxiety Inventory (STAI) scale among dyslexic students. Our results are also in accord with the findings of several other studies that suggest that children with reading difficulties are significantly more depressed than their peers.^7^ ^,^ ^9^ In response to school activities, students with learning disabilities can develop as many unpleasant emotions (i.e., sadness, anger, disappointment, despair, shame, and guilt). Similarly, the stress generated by the situation of powerlessness and ineffectiveness at school can lead to an increased risk of developing neuropsychiatric disorders, such as depression and dementia.^27^ In contrast, among children with learning difficulties such as dyslexics, the alteration of attentional processes and working memory (which are already overloaded in relation to the non-automated reading task) by pathological anxiety aggravates information processing dysfunction.^28^ It goes without saying that the feeling of competence is one of the main drivers of a child’s psychological development and what structures his/her identity as a student, i.e., the way he/she perceives himself/herself as an actor in the learning process of academic knowledge and knowledge in general. This feeling is very dependent on the child’s confidence in his/her own resources and confidence in the support he/she can receive from the environment, ultimately on his/her self-esteem.^29^

We can also compare our results to those obtained in a study conducted among young dyslexics aged 8–16 years old, which alerts us to the high level of depression in these young people.^30^ The same study reported that one out of two dyslexics has lower school self-esteem than the average of the “control” group. In another study, low self-esteem was found in dyslexic children between the ages of 8 and 15.^31^ The self-esteem level was inversely associated with the severity of anxiety and depression disorders. The analysis of the data collected in relation to the Self-Esteem assessment also provides results that differ according to the reading level. Thus, unlike dyslexics, in the group without reading difficulties, boys had significantly higher self-esteem than girls in the family domain and significantly very low self-esteem in the school component of the inventory. Numerous studies have shown that average self-esteem scores are higher for boys, who tend to overestimate and overvalue their abilities than for girls, especially during the first part of adolescence.^32^ However, in other studies, differences between girls and boys are reported only in self-esteem related to certain areas of functioning such as sports and athletics,^33^ ^,^ ^34^ not in others such as social and academic skills.^34^ A child may therefore present high self-esteem in one or more areas (e.g., physical appearance, popularity, and conformity) but self-evaluate negatively in other areas (e.g., academic achievement and athletic skills). In the case of our study, the source of the observed difference in favor of boys would be the social environment, particularly the family environment. Thus, parents tend to encourage their boys to defend their interests and assert their personality more than they do in the case of girls. They tolerate more shyness in girls and encourage them to be docile, obedient, and coquettish behavior that is not conducive to the development of stable and strong self-esteem.^35^ The importance given to each of the life domains represents another cognitive factor that also intervenes in the differences in self-esteem noted between girls and boys.^35^

Concerning the intrication of dyslexia and internalized psychiatric disorders, we found that 82% of “clinical” group subjects had a “low to very low” self-esteem (score≤30). For anxiety symptomatology and depressive mood, the percentages of the “severe to very severe” intensity are 72.7 and 94%, respectively. Comparing these values with those obtained for the “control” group (17, 19, and 53%, respectively), we believe that the association between the two categories of “pathologies” is included in real comorbidity in the neurobiological sense of the term. However, even in the absence of genetic links, due to the persistent nature of dyslexia, the complex emotional situation of dyslexics may be secondary to the feelings of weakness and incompetence generated by this cognitive “disability.” In fact, facing a demanding education system that does not yet recognize dyslexia as a complex disorder and the misunderstanding of teachers who ignore this “handicap,” because it was not part of their training, provokes a daily confrontation with failure. However, it must be emphasized that there is no direct cause-and-effect relationship between dyslexia and emotions. The potential emotional distress is not generated by the reading difficulty itself, but by the way, society treats the difference marking the dyslexic child. As studies point out, students who attend dyslexia-friendly schools or inclusive schools that provide integration features have similar levels of stress and self-esteem as their non-dyslexic peers, since their unique aspects are understood and their educational needs are met.^36^ ^,^ ^37^ It is therefore interesting to note that the emotional difficulties frequently recorded in dyslexics are not the result of neurological dysfunction, but they are multifaceted phenomena, pervaded by socio-historic and cultural aspects. In the Moroccan context, the majority of parents and teachers interpret the performance of learners who do not succeed in school as a lack of motivation, laziness, and indifference. These judgments are reasonable because families are not made aware of the specific disorders of which dyslexia is a part. In addition, in many cases, school failure, which is an inevitable consequence of specific learning disabilities, is a shame and a stigma for families. In other cases, people and neighbors empathize with families who have children who are not able to succeed in their end-of-year examinations. These are some of the factors that will have a significant impact and repercussion on the emotional well-being of learners with dyslexia. In addition, the frustration generated by the unrewarded effort and narcissistic injury of permanent peer comparison, in overcrowded ordinary classes, causes a remarkable alteration in self-esteem and a disturbing state of anxiety and depression. These psychopathological symptoms will in return have a negative impact on the academic performance.

It emerged from this study that dyslexic children and adolescents differentiated from their peers without dyslexia by significantly impaired the overall self-esteem and much more frequent anxiety and depression symptoms. These results reinforce the conclusions of the overwhelming majority of studies, dealing with the same issue, which described pupils with specific written language disorders as more vulnerable to psychopathological difficulties. In conclusion, the results of this study encourage us to emphasize the need for early prevention of the psycho-affective difficulties of subjects suffering from this invisible cognitive “handicap.” It highlights the importance of addressing specific learning disabilities, in general, and dyslexia, in particular, as a real public health problem. This urges health professionals and educational sector authorities to provide appropriate care for dyslexic students. Hence, the need to continue this type of study in the Arabic-speaking scientific landscape to deepen the knowledge around these difficulties and to identify the factors generating psychological distress in children with reading disorders. However, it should be pointed out that the fact of basing ourselves only on standardized scales, which do not always correspond to the observed reality, is an element that could be considered as a major limitation of our study. A clinical evaluation based on the lived experiences of schoolchildren, by multidisciplinary school health teams, is an urgent necessity.
